# Measurement of Optical Properties of CH_3_NH_3_PbX_3_ (X = Br, I) Single Crystals Using Terahertz Time-Domain Spectroscopy

**DOI:** 10.3390/ma16020610

**Published:** 2023-01-08

**Authors:** Srinivasa Rao Konda, Yucai Lin, Rahul A. Rajan, Weili Yu, Wei Li

**Affiliations:** GPL Photonics Laboratory, State Key Laboratory of Luminescence and Applications, Changchun Institute of Optics, Fine Mechanics and Physics, Chinese Academy of Sciences, Changchun 130033, China

**Keywords:** THz time-domain spectroscopy, MAPbBr_3_, MAPbI_3_, THz optical properties, THz generation

## Abstract

Organometallic lead bromide and iodide perovskite single crystals (PSCs) are potential candidates for terahertz applications. Herein, we performed terahertz time-domain spectroscopy (THz-TDS) in the frequency range of 0.1–3.0 THz on different thicknesses of MAPbBr_3_ (0.3, 0.6, and 0.8 mm) and MAPbI_3_ (0.6, 0.8, 0.9, 1.3, and 2.3 mm). The measurements were carried out with respect to the position (along the focal area), azimuthal rotation of the PSCs, and incidence angles of the reference THz pulse on the PSCs’ surface. Based on the transmitted THz pulses from PSCs from the above measurements, we calculated the real and imaginary parts of the refractive index, dielectric constants, absorption coefficients, and dark conductivity. These optical parameters tend to increase with decreases in the PSCs’ thicknesses. The transmission spectra of the terahertz electric field indicate that the measured optical properties do not vary significantly with the position and orientation of PSCs. The real parts of the refractive index and dielectric constants are higher than the imaginary values for both PSCs. On the other hand, a slight blueshift in the optical phonon vibrations corresponding to Pb-Br/I-Pb and Pb-Br/I bonds is observed with an increase in thickness. Interestingly, the phonon vibrations do not vary with the incidence angle of the THz pulses on the same crystal’s surface. The optical parameters based on THz-TDS reveal that the PSCs satisfy the requirement for tunable THz devices which need suitable, sensitive, and stable absorption properties between 0.1 and 3 THz.

## 1. Introduction

Terahertz (THz) radiation has spectral bands with frequencies ranging from 0.1–10 THz. Recently, in our laboratory we initiated the THz generation from air plasma using a two-color pump (800 nm+ 400 nm wavelengths) with femtosecond amplifier pulses and optimized the applications of THz radiation using time-domain THz spectroscopy (THz-TDS). Air plasma is one of the potential sources for THz generation [1,2]. In addition, several research groups explored the THz generation from numerous materials with different laser sources and a variety of THz applications using THz-TDS in optics, photonics, biomedicine, and imaging [3,4,5,6,7,8,9]. The main advantage of the THz-TDS technique is that one can measure the frequency-dependent complex refractive index of the material. In addition, it can help to find the real and imaginary parts of dielectric constants, absorption coefficients, and conductivity, which are crucial in understanding the fundamental physics that ultimately defines the material’s performances in the THz domain.

In recent years, the organic–inorganic halide perovskite MAPbX_3_ (X = Cl, Br, I) obtained wide attention for its optoelectronic and photonic properties [10,11,12,13]; however, limited attention has been paid to the investigation of the nonlinear optical properties associated with the perovskite single crystals (PSCs) in the THz domain. Determining the nonlinear optical constants such as the refractive index, absorption coefficients, and dielectric constants at the THz domain is highly important for the design and development of perovskite-based THz devices. Recently, several research groups studied the optical properties of organic–inorganic halide perovskites in the THz range [14,15,16]. For example, Nayak et al. studied the elemental, optical, and THz-TDS studies on methyl p-hydroxybenzoate SC for THz applications [17]. Chanana et al. demonstrated ultrafast frequency-agile terahertz metamaterial devices using polycrystalline perovskite thin films deposited on metamaterial structures [14]. Zhao et al. reviewed some of the most representative works in halide perovskite research using THz-TDS and time-resolved terahertz spectroscopy and revealed the importance of ultrafast terahertz techniques [16]. Xia et al. observed optical phonons associated with Pb–I stretching in both MAPbI_3_ SCs and polycrystalline thin films as a function of temperature by measuring their terahertz conductivity spectra with and without photoexcitation [18]. Maeng et al. explored the unique phonon modes of a MAPbBr_3_ hybrid perovskite film towards the novel THz-based application, mentioned that “if we can control the THz-wave absorption property in organic-inorganic perovskite thin film on a flexible substrate, we can eventually achieve flexible THz-wave sensing, modulation, and imaging devices” [19], and simulated IR spectra for MAPbI_3_ [20]. Most of the abovementioned studies paid attention to the dependence of THz optical properties in the case of thin films.

However, novel strategies to enhance the THz properties are still lacking. In this research, we paid attention to PSCs with varying thicknesses and the incident angle of THz pulses to the crystals’ surface. We further investigated the THz optical properties of the studied PSCs, such as refractive index, absorption coefficients, dielectric constants, and dark conductivity (measured by without photoexcitation) in the THz domain. On the other hand, the behavior of phonon frequencies in the MAPbBr_3_ and MAPbI_3_ owing to the Pb-X-Pb, Pb-X vibrations were studied. We observed that the sample thickness leads to variations in the transmitted THz pulses and causes a small blueshift in the phonon vibrations of Pb-X-Pb, Pb-X bonds. In addition, we measured the THz optical properties of the studied PSCs with the change in incidence angle on the single crystal surface between 0–45°. Interestingly, we observed that the incidence angle on the single crystal surface does not significantly affect the phonon vibrations. As per our knowledge, the current work is the first report on THz optical properties with varying thicknesses of bulk PSCs.

Particularly, for the generation of THz radiation from air plasma, we initially controlled the phase difference between fundamental and second harmonic wavelengths to achieve the higher THz peak amplitude and bandwidth for the reference pulse and investigated the transmitted THz from PSCs. Further, at fixed THz reference pulses, we measured the transmitted profiles of PSCs by changing their azimuthal orientation and movement along the focal plane of THz pulses. The experimental observations indicate that the PSCs have unique absorption bands in the THz domain compared with earlier reported works on these PSCs [18,19]. However, the PSCs’ thickness is the key factor in enhancing the amplitude of transmitted THz pulse and measured optical parameters in the THz domain, having higher values for smaller-thickness PSCs. Thus, the current study reveals the unique THz transmission properties of MAPbX3 (X = Br, I) PSCs with varying thicknesses, implying the advantages of smaller-thickness PSCs for applications in THz devices [19].

## 2. Experimental and Theoretical Details

### 2.1. Materials’ Synthesis

**Figure 1:**
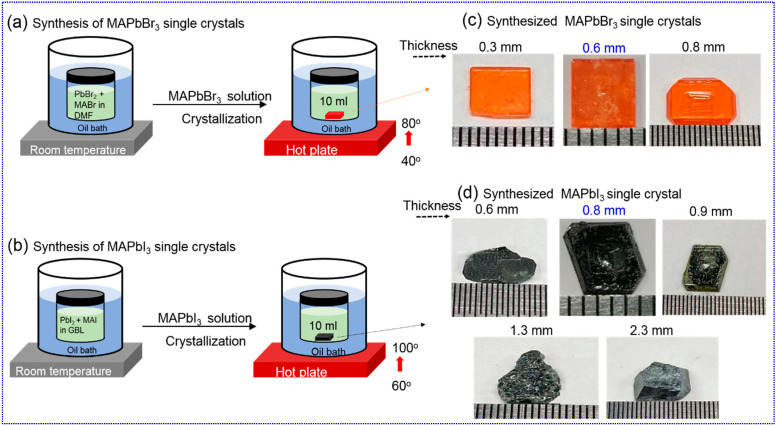
Synthesis schematic of (**a**) MAPbBr_3_ and (**b**) MAPbI_3_ single crystals using inverse temperature crystallization method. Images of as-synthesized (**c**) 0.3, 0.6, and 0.8 mm MAPbBr_3_ single crystals and (**d**) 0.6, 0.8, 0.9, 1.3, and 2.3 mm MAPbI_3_ single crystals.

*The growth process of MAPbBr_3_ and MAPbI_3_ single crystals:* Figure 1a,b show the schematic of the step-by-step preparation of MAPbBr_3_ and MAPbI_3_ PSCs, respectively. In brief, the inverse temperature crystallization (ITC) was used to grow MAPbBr_3_ and MAPbI_3_ single crystals [21]. First, the 1M precursor solution (PS) was prepared by taking 1:1 equivalent of PbBr_2_: MABr in N, N-dimethylformamide (DMF) at room temperature for MAPbBr_3_ and 1:1 of PbI_2_: MAI in Gamma butyrolactone (GBL) at 60 °C for MAPbI_3_, respectively. Subsequently, both solutions were stirred for 2 h until a clear solution formed. Afterwards, a vial with 10 mL MAPbBr_3_/MAPbI_3_ PS was kept in an oil bath undisturbed at 40 °C/60 °C for 2 h, and then the oil temperature gradually increased from 40 °C to 80 °C (MAPbBr_3_) and 60 °C to 100 °C (MAPbI_3_) until individual single crystals were formed. The obtained single crystals of MAPbBr_3_ (thickness: 0.3, 0.6, and 0.8 mm) and MAPbI_3_ (thickness: 0.6, 0.8, 0.9, 1.3, and 2.3 mm) are shown in Figure 1c,d, respectively.

### 2.2. Experimental Details

The excitation wavelengths of 473 nm and 532 nm were used to measure the MAPbBr_3_ and MAPbI_3_ photoluminescence spectra (HORIBA Scientific Raman spectrometer, Kyoto, Japan), respectively, with an intensity of 0.1% at room temperature.

The X-ray diffraction data for PSCs and their powder form were measured using an XRD instrument (Bruker Corporation, D8 FOCUS, Karlsruhe, Germany).

**Figure 2:**
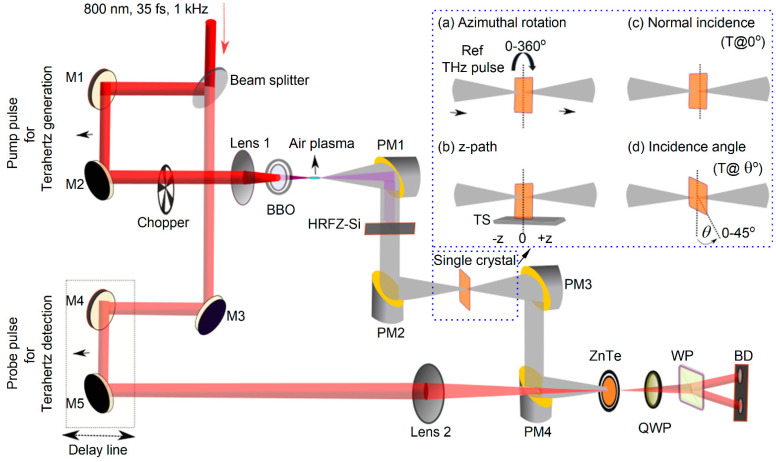
THz-TDS experimental layout. Beam splitter R:T (90:10), BBO (type 1, 0.2 mm), ZnTe (110, 1 mm thick), lens 1 (f = 150 mm), lens 2 (f =300 mm), M1–M5: mirrors, PM1–PM4: off-axis parabolic mirrors, TS: translation stage, QWP: quarter waveplate, WP: Wollaston prism, and BD: balanced diodes. Insets show at normal incidence of input THz pulse (**a**) single crystal azimuthal orientation and (**b**) single crystal moved along the z-path. Representation of (**c**) incident THz pulse at normal incidence to the single crystals and (**d**) change of incidence angle on the single crystal surface.

*Experimental details of THz-TDS:*Figure 2 shows the indigenously built THz-TDS system in ambient conditions in the range of 0.1 to 3.0 THz. A Ti: Sapphire laser delivered 800 nm, 35 fs, 1 kHz pulses. A beam splitter (90:10) was used to split the output beam into pump (generation arm) and probe (detection arm) sources. We fixed the pump’s laser pulse energy at 1.5 mJ and 3 µJ for probe pulses. The pump and probe pulses were focused by a spherical lens of 150 mm and 300 mm focal lengths, which have corresponding beam waists around 16.5 µm and 30.75 µm, respectively. A 200 µm thick type 1 BBO crystal was placed on a rotating mount (0–360°) and kept at a 90 mm distance from the spherical lens position. The generated THz radiation from air plasma was guided by four gold-coated half-axis parabolic mirrors (PM) until the THz signal was focused on the ZnTe (110) crystal (EKSMA optics) having 1 mm thickness. THz radiation was detected using an electro-optical sampling technique consisting of a ZnTe crystal, quarter waveplate, Wollaston prism, and balanced photodiodes. The residual input laser was blocked by high-resistivity float zone silicon (HRFZ-Si) after the PM1. The PSCs were placed on a rotating mount and moved along the focal plane of PM2 using the translational stage for position-dependent THz-TDS measurements. The output of the balanced diode signal was connected to the lock-in amplifier (Stanford Research Systems, model no. SR830). The pump pulse was chopped at 500 Hz using a mechanical optical chopper used as a reference to the lock-in amplifier. The LabVIEW program controlled whole data acquisition. Initially, the THz signal generated from the air plasma using the femtosecond laser source was transmitted through dry air (laboratory environment: room temperature 20 °C and humidity 5%), considered a reference signal in terms of a time-varying electric field. The attained THz temporal waveforms were Fourier Transformed applying numerical FFT, and the achieved complex values of $\psi_{SC}\left(\omega \right)$ were divided by $\psi_{ref}\left(\omega \right)$. The theoretical equations are given in Section 2.3.

### 2.3. Theoretical Equations for THz-Based Optical Parameters

The complex Fast Fourier Transform (FFT) of PSCs’ THz transmission with respect to the reference is given by

(1) $$\frac{\psi_{SC}}{\psi_{ref}}=\rho \left(\omega \right)e^{-i\phi \left(\omega \right)}$$

where $\psi_{SC}$ and $\psi_{ref}$ are the complex FFT of the single crystal and reference THz pulse, respectively, $\phi \left(\omega \right)$ is the phase difference, and $\rho \left(\omega \right)$ is the ratio of peak amplitudes of single crystal and reference pulses. The real refractive index (*n*_SC_) and imaginary refractive index (*k*_SC_) can be written in terms of ϕ(ω) and $\rho \left(\omega \right)$ as follows [22,23]:

(2) $$n_{SC}\left(\omega \right)=\phi \left(\omega \right)\frac{c_{o}}{\omega d}+1$$

(3) $$k_{SC}\left(\omega \right)=\ln\left(\frac{4n_{SC}\left(\omega \right)}{\rho \left(\omega \right){\left(n_{SC}\left(\omega \right)+1\right)}^{2}}\right)\frac{c_{o}}{\omega d}$$

where *c*_o_ is the speed of light and *d* is the single crystal thickness. From the imaginary part of the refractive index, one can determine the absorption coefficient *α* (cm^−1^) as

(4) $$\alpha \left(\omega \right)=\frac{2}{d}\ln\frac{4n_{SC}\left(\omega \right)}{\rho \left(\omega \right){\left(n_{SC}\left(\omega \right)+1\right)}^{2}}$$

In addition, the complex dielectric constant can be generalized like Maxwell’s relation based on the complex refractive index relation [24]:

(5) $$\epsilon ={\left[n(\omega )\right]}^{2}$$

Which can be stated as

(6) $${n(\omega )}^{2}-{k\left(\omega \right)}^{2}-2jn\left(\omega \right)k\left(\omega \right)=\epsilon^{\prime }-{i\epsilon }^{\prime \prime }$$

Here, $\epsilon^{\prime }$ and $\epsilon^{\prime \prime }$ are the real and imaginary dielectric constants determined from the following equations, where

(7) $$\epsilon_{SC}^{\prime }\left(\omega \right)={n_{SC}(\omega )}^{2}-{\left(\frac{c_{o}\alpha (\omega )}{2\omega }\right)}^{2}\mathrm{and}\;\epsilon_{SC}^{”}\left(\omega \right)=2n_{SC}\left(\omega \right)k_{SC}(\omega )$$

Finally, the dielectric constant can be expressed as

(8) $$\epsilon_{SC}\left(\omega \right)=\epsilon_{SC}^{\prime }\left(\omega \right)+\epsilon_{SC}^{\prime \prime }\left(\omega \right)$$

The ‘dark’ conductivity (*σ_dark_*(ω)) was calculated directly from the experimental THz transmission by considering the real part of the refractive index spectra [18,25,26], which is given by

(9) $$\sigma_{dark}\left(\omega \right)=\frac{1}{Zd}(1+n_{sc})\left(\frac{1}{T(\omega )}-1\right)$$

where *Z* = 377 Ω is the free space wave impedance and $T\left(\omega \right)=T_{sc}\left(\omega \right)/T_{ref}\left(\omega \right)$ is the transmittance of a single crystal terahertz electric field.

## 3. Results and Discussions

### 3.1. Structural Characterization and Optical Properties of PSCs

**Figure 3:**
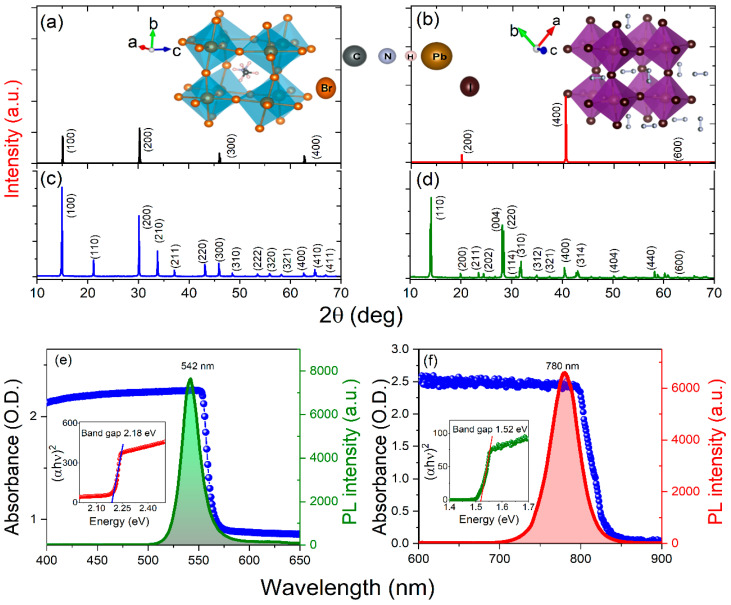
XRD analysis of (**a**) MAPbBr_3_ and (**b**) MAPbI_3_ PSCs (inset: as-synthesized PSCs) and (**c**,**d**) powders. (**e**,**f**) Steady-state UV-Visible absorption and photoluminescence, respectively. Insets: Tauc plots exhibit the extrapolated optical band gaps.

To know the optical bandgap and crystallinity of the as-synthesized PSCs, we conducted steady-state UV-Visible absorption, photoluminescence, and XRD measurements. The optical and morphological property of PSCs depend on the synthesis procedure [20]. However, in the present work, we synthesized different thicknesses of MAPbBr_3_ and MAPbI_3_ PSCs using the same ITC method. Therefore, there might be no chance to obtain different types of PSCs during the manufacturing (synthesis) processes. Therefore, we could expect that the crystallinity, optical band gap, and PL emission properties will be affected by the different thicknesses of the samples. Figure 3a,b show the XRD data for 0.6 mm -MAPbBr_3_ and 0.8 mm- MAPbI_3_, the corresponding diffraction angles 2θ (miller indices) of 15.04 (100), 30.42 (200), and 46.10 (300) degrees, and 20.23 (200), 40.43 (400), and 62.12(600) degrees, which confirm that MAPbBr_3_ and MAPbI_3_ possess cubic and tetragonal crystal structure, respectively. The Figure 3a,b inset shows cubic MAPbBr_3_ and tetragonal MAPbI_3_ molecular structures. Figure 3c,d show the XRD pattern for powder PSCs, and the corresponding miller indices are labeled in the graphs.

Figure 3e,f show the steady-state absorption properties and PL emission for MAPbBr_3_ and MAPbI_3,_ respectively. The corresponding inset shows the Tauc plots exhibiting the extrapolated optical band gap (*E*_g_) of each PSC. The measured bandgap of MAPbBr_3_ is *E*_g_ = 2.18 eV, and MAPbI_3_ is *E*_g_ = 1.52 eV. In the PL spectra of MAPbBr_3_ and MAPbI_3_ around 542 nm and 780 nm, peak positions were achieved. These values are consistent with the earlier synthesized crystals using the ITC method [21]. The higher excitation wavelengths lead to a redshift in the PL peak position due to changes in the nonlinear absorption process, which depends on the optical photon energy [13,27].

### 3.2. THz-TDS of PSCs

THz radiation supports this capability to explore the material properties by choosing absorption associated with intermolecular interfaces. THz-TDS is one of the valuable techniques in ascertaining the response of a material in the far-infrared region to near microwaves of electromagnetic radiation. The optical properties of the PSCs, i.e., MAPbBr_3_ and MAPbI_3_ in the THz regime, were measured based on experimental data obtained from THz-TDS measurements using the theoretical equations shown in Section 2.3.

#### 3.2.1. Optimization of Reference THz Pulse, THz-TDS of PSCs for Azimuthal Rotation, and Movement along the Z-Path

**Figure 4:**
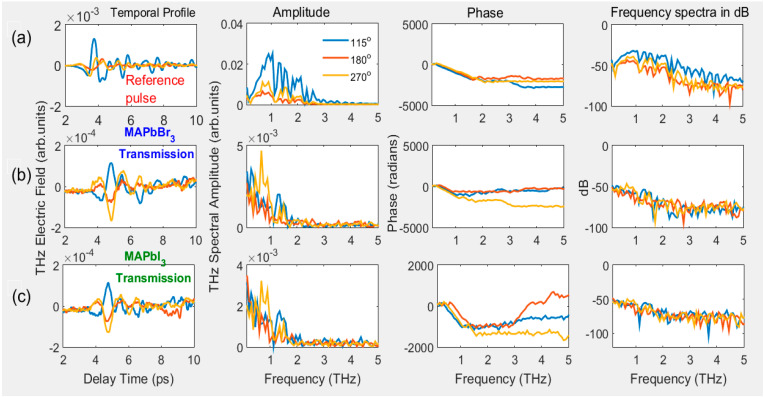
Generated THz pulse temporal profile, amplitude, phase, and frequency spectra (**a**) at rotational angles of BBO 115°, 180°, and 270° (measured between 0–360°, with 5° interval but presented only at three angles). THz transmission from (**b**) MAPbBr_3_ and (**c**) MAPbI_3_ PSCs using refence THz pulses from (**a**). The PSCs kept at the focus position of THz pulses from PM_2_.

**Figure 5:**
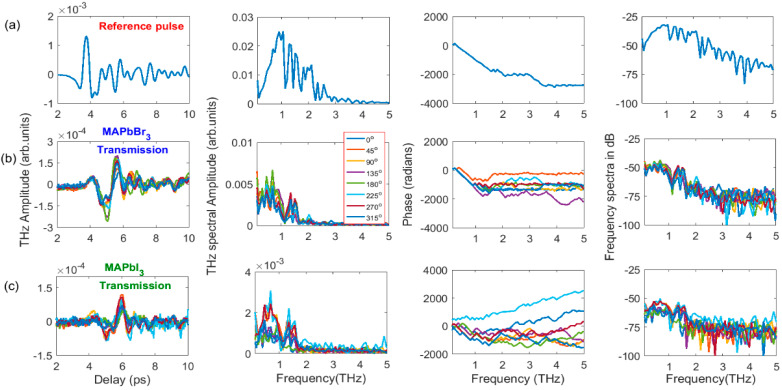
Temporal profile, amplitude, phase, and frequency spectra of (**a**) reference pulse at rotational angles of BBO 115° and corresponding THz transmission from (**b**) MAPbBr_3_ and (**c**) MAPbI_3_ PSCs concerning their azimuthal orientation.

**Figure 6:**
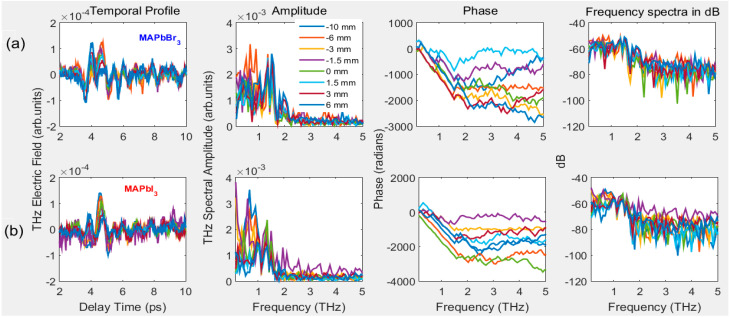
Temporal profile, amplitude, phase, and frequency spectra of (**a**) MAPbBr_3_ and (**b**) MAPbI_3_ PSCs. The samples moved along the *z*-axis near the focal area of PM2 between -10 mm and 10 mm.

Prior to extracting optical parameters, for the source of THz pulses, the ultrashort two-color femtoseconds pulses were focused on the ambient air. The resulting THz signal was optimized by controlling the phase difference between fundamental and second harmonic wavelengths employing the azimuthal orientation of the type 1 BBO crystal, as shown in Figure 4. Further, we used an optimized reference THz pulse, which is normally incident (T@0°) to the PSCs’ surface, and after that PSCs were azimuthally oriented between 0–360° and moved along the *z*-axis (−10 mm to 10 mm). The corresponding transmissions of THz pulses from PSCs are shown in Figure 5 and Figure 6, respectively.

**Figure 7:**
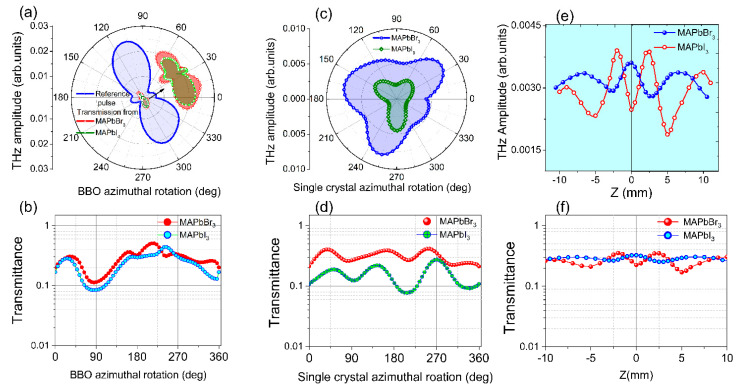
The peak amplitudes of (**a**) reference THz pulse and transmission from PSCs’ (**b**) measured transmittance. At optimized THz reference pulse, transmitted THz peak amplitudes, transmittance from PSCs w.r. to (**c**,**d**) azimuthal rotation (**e**,**f**) moved along the *z*-axis near the focal plane of MAPbBr_3_ and MAPbI_3_ single crystals, respectively.

In Figure 4a, the first panel shows the generated THz pulses from air plasma at BBO rotation angles of 115, 180, and 270°, and panels 2–4 show, after converting the THz electric field to the FFT frequency spectra, the corresponding amplitude, phase, and frequency spectra in dB, respectively. Figure 4b,c depict the related THz transmission from PSCs at a normal incidence of THz pulse. The generated THz from air plasma indicates that the phase difference between the 800 nm and 400 nm wavelengths significantly affects the peak amplitude. In the current study, we achieved a higher THz signal at 115° and 315°. We show the obtained THz temporal profiles at three angles in Figure 4a; we measured the THz signal at each 5° difference in the 0–360° range. For all the azimuthal angles of BBO, the obtained peak amplitudes of generated THz pulses are shown in Figure 7a, which also consists of the peak amplitudes of THz transmission from both PSCs corresponding to the input reference THz pulses.

The change in the rotational angle of BBO leads to varying THz amplitudes, whereas the phase remained the same within the valid range of THz frequencies. Correspondingly, the PSCs possess unique transmission properties, except for a small difference in the amplitude of the transmitted pulse (see Figure 4b,c). The amplitudes of transmitted THz pulses from PSCs are proportional to the reference THz pulse amplitude, which is shown in the inset of Figure 7a. However, the measured transmittance of PSCs for their reference pulses is shown in Figure 7d. In this case, one can clearly see that both PSCs possess almost identical THz transmission properties, due to their similarity of molecular chemical formula MAPbX_3_(X = I, Br). The transmittance is higher for MAPbBr_3_ than MAPbI_3,_ probably due to the 0.2 mm lower thickness.

Figure 4a shows the orientation of BBO between 0–360°, and shows the maximum THz amplitude near 115°; this angle is consistent with earlier work [28]. THz transmission from PSCs was measured at each azimuthal angle between 0–360° (some of the angles are shown in Figure 5b,c). The data shown in Figure 5b imply that the temporal profiles and THz amplitude look similar with the change in the azimuthal angle, which leads to no variation in the measured transmittance (Figure 7d). However, among the small indicated changes, the crystals possess maximum transmission peaks around 35, 165, and 255 (MAPbBr_3_) and 55, 145, and 275° (MAPbI_3_).

In addition, we also measured the THz transmission of PSCs by moving them in the focal plane along the *z*-axis. The obtained temporal profiles of the amplitude, phase, and frequency spectra in dB are shown in Figure 6a,b for MAPbBr_3_ and MAPbI_3_ crystals, respectively. In this case, like the azimuthal orientation of PSCs, no difference is observed in the shape of temporal profiles, except for a small difference in their peak amplitude values. In Figure 7e,f, we present the THz amplitude and measured transmittance concerning changes in the positions of PSCs along the *z*-axis from the −10 mm to 10 mm range corresponding to the focal plane. It is observed that the peak amplitude curves are symmetrical regarding the focal position of THz pulses, with peak amplitude changes between 0.002 to 0.004 in the arbitrary unit range. Suppose we consider the transmittance to the reference THz pulse shown in Figure 7f. At almost all positions, the PSCs possessed similar transmittance with small variation; however, within this range, MAPbBr_3_ and MAPbI_3_ possess maximum and minimum transmittance at the focus position, respectively.

The measured transmittance at different conditions revealed that the PSCs have unique THz transmission properties without the excitation of any pump wavelength. One can obtain the conductivity, mobility, and lifetime of charge carriers in the case of the photoexcitation of PSCs with an optical pump and THz probe (time-resolved THz spectroscopy). However, in the present work, we limited our study to THz-TDS, which was enough to extract information about complex refractive index and dielectric constants, absorption coefficients, and dark conductivity from the transmitted THz electric field spectra of PSCs.

#### 3.2.2. Measurements of THz Optical Properties with Varying Thicknesses of PSCs

**Figure 8:**
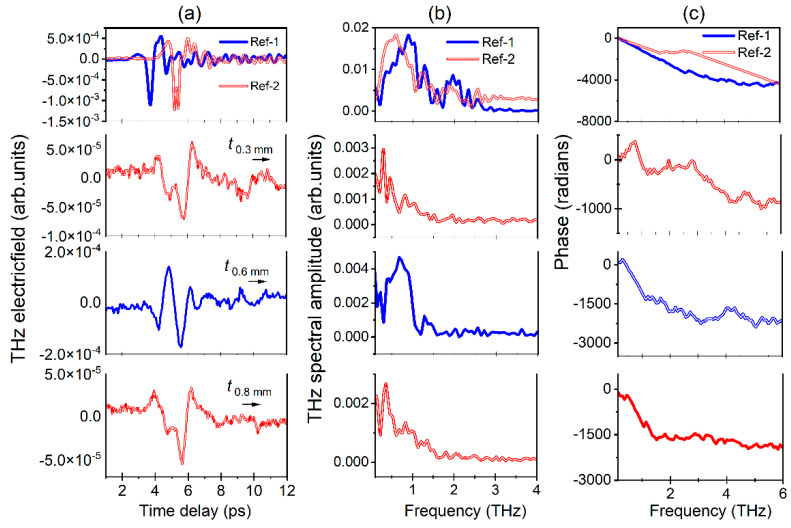
THz temporal profiles (**a**, upper panel) of reference 1 and reference 2 measured at two different dates at BBO rotation angle 115° the reference 2 pulse after transmission from MAPbBr_3_ (0.3 and 0.8 mm) and reference pulse 1 transmitted from 0.6 mm MAPbBr_3_. After FFT: (**b**) spectral amplitude and (**c**) phase, respectively.

**Figure 9:**
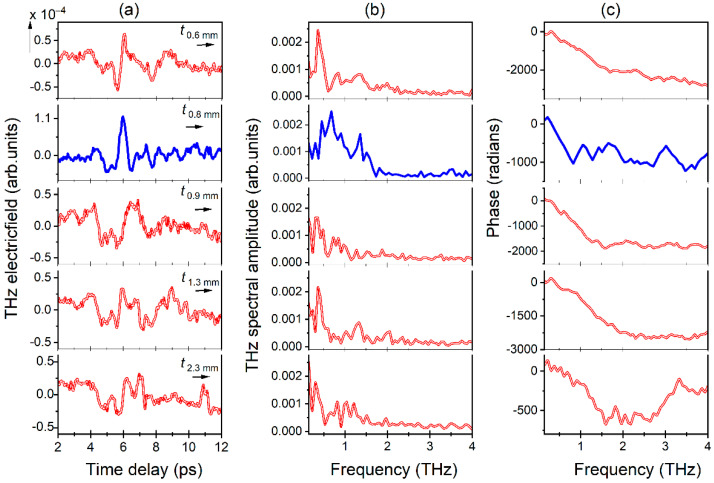
THz temporal profiles (**a**, second panel) using reference 1 MAPbI_3_ (0.8 mm), and reference 2 measured after transmission from 0.6, 0.9, 1.3, and 2.3 mm MAPbI_3_ (a, 1,3,4,5 panels top to bottom) PSCs. After FFT: (**b**) spectral amplitude; (**c**) phase of transmitted THz pulse corresponding thickness of MAPbI_3_ shown in (**a**).

The first panel in Figure 8a shows the temporal profiles of two reference pulses obtained from a BBO rotation of 115° in two measurements, whereas the panels from two to four represent the transmission of reference pulses from 0.3, 0.6, and 0.8 mm, respectively. Figure 8b,c show the amplitude and phase obtained from the FFT of temporal spectra shown in Figure 8a. Similarly, the second panel in Figure 9a shows the temporal profile obtained from the transmission of reference pulse 1 from the 0.8 mm thick MAPbI_3_, whereas the panels one and from three to five represent the transmission of reference pulse 2 from 0.6, 0.9, 1.3, and 2.3 mm, respectively.

Figure 9b,c show the amplitude and phase obtained from the FFT of temporal spectra shown in Figure 9a. It is observed that the cut-off frequency was achieved up to 3.0 THz for both main reference pulses. In the first set of measurements, the MAPbX_3_ PSCs are placed normally to the incidence angle of the THZ pulses (@T 0°). The data shown in Figure 8 and Figure 9 clearly reveal that the smaller-thickness PSCs possess higher transmission, and as a result higher THz peak amplitude is achieved. The temporal profiles also seem quite similar for each SC. The transmitted THz pulses from PSCs show an extended time delay compared to the reference pulse, which is clearer in the case of first set of crystals. For example, in the case of MAPbI_3_ (0.8 mm) and MAPbBr_3_ (0.6 mm), there are higher THz peak amplitude positions located at 5.97 ps and 5.57 ps, compared to the reference at 3.69 ps. Figure 8b,c and Figure 9b,c show the amplitude and phase for MAPbBr_3_ and MAPbI_3_, which are essential entities in measuring THz optical parameters using THz-TDS.

**Figure 10:**
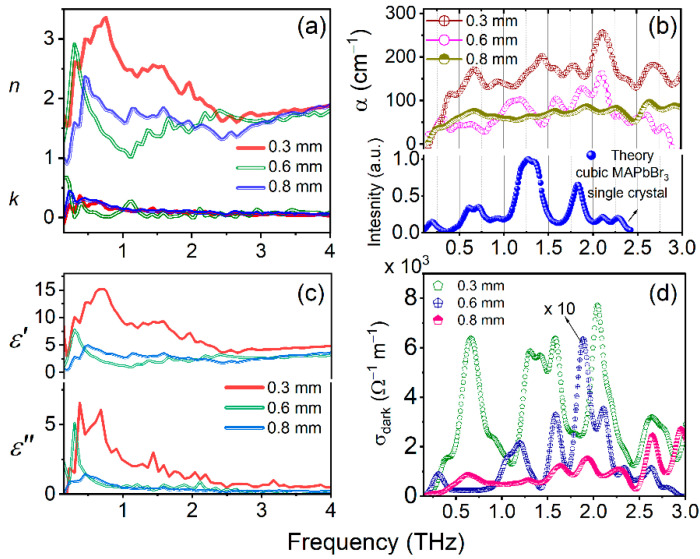
(**a**) Real refractive index (n), imaginary refractive index (k); (**b**) absorption coefficient (α), bottom panel theoretically calculated IR spectrum for cubic MAPbBr_3_ single crystal (data adapted from Ref. [19], with permission from Springer nature). (**c**) Real dielectric constant (ε′), imaginary dielectric constant (ε″), and (**d**) dark conductivity of various-thickness MAPbBr_3_ PSCs measured based on THZ -TDS.

**Figure 11:**
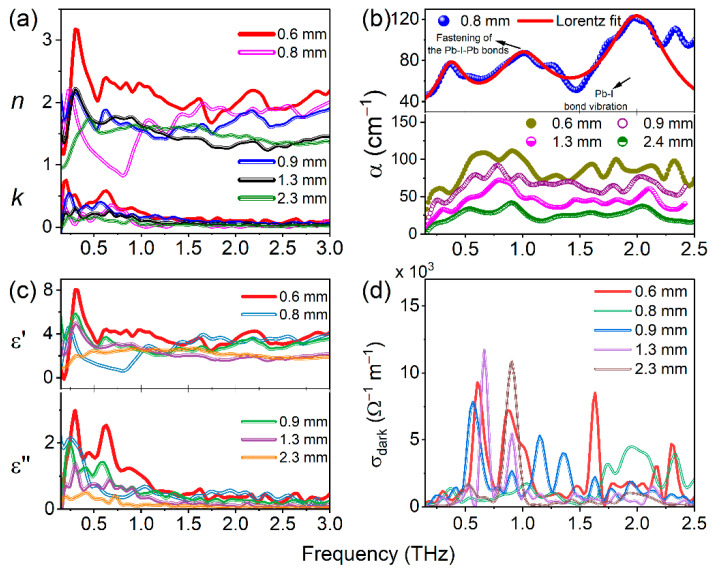
(**a**) Real refractive index (n), imaginary refractive index (k); (**b**) absorption coefficient (α), upper panel solid line represents Lorentz peak fit for 0.8 mm MAPbI_3_ experimental α data; (**c**) real dielectric constant (ε’), imaginary dielectric constant (ε”); and (**d**) dark conductivity of various thickness MAPbI_3_ PSCs measured based on THZ-TDS.

It was observed that the reference THz pulse band width was decreased after transmission of PSCs due to their absorption properties. The corresponding cut-off achieved nearly 1.75 THz for both series of crystals. Similarly, up to this frequency almost all the studied PSCs possessed a linear decrease (Figure 8c and Figure 9c). The complex refractive index (i.e., real and imaginary), absorption coefficient (α), complex dielectric constant, and dark conductivity of the MAPbX_3_ (X = I, Br) PSCs with varying thicknesses are shown in Figure 10a–d and Figure 11a–d, respectively. These parameters were calculated from the data shown in Figure 8 and Figure 9 using the corresponding Equations (2), (3), (4), and (7), respectively. The refractive index of PSCs shows nonlinear behavior in the THz frequencies (Figure 10a and Figure 11a) with respect to different thicknesses of the PSCs. For example, it is observed that the real part of the index of refraction (*n*_SC_) varies between 1.09506–2.91397 for MAPbBr_3_ (0.6 mm) and 1.41693–2.19141 for MAPbI_3_ (0.8 mm). The maximum real refractive indices 2.91397 and 2.19141 were achieved at 0.30 THz and 0.22 THz frequencies for MAPbBr_3_ and MAPbI_3_, respectively, and after this maximum position they exponentially dropped by up to 1 around 1.05 THz and after, which shows the consistency behind the two THz pulses. Generally, the refractive index of the materials is independent of their thickness. The higher thickness increases the density of materials, which leads to decreases in the transmission of the THz pulse. As one can understand, in the THz domain the refractive index of the material was calculated from the phase difference of the transmitted THz pulse from the single crystals and reference pulse, including the thickness of the single crystals with respect to THz frequencies (as per Equation (2)). In this regard, the phase difference leads to variation in the refractive index, which shows nonlinear behavior with respect to the THz frequency range. Consequently, the higher values of refractive indices for these PSCs allow light rays to bend more within the material, which helps in lowering the thickness and results in less weight. Further, the samples’ lower weight and thickness can be deposited in metamaterial structures and could be useful for various THz devices [14]. As we mentioned in the introduction, Xia et al. demonstrated that a 10 mm thick MAPbI_3_ single crystal lowers the THz transmission. However, selecting the lower thickness of PSCs could enhance the THz transmission [18].

In the case of both PSCs, the real parts of the refractive index and dielectric constants are higher than the imaginary parts. The curve shapes of real dielectric constants (Figure 10c and Figure 11c) are very similar to the real parts of the refractive indices (Figure 10a and Figure 11a) because they were obtained using *n*_SC_ (Equation (7)). The PSCs possess higher values of dielectric constants, shown in Figure 10c and Figure 11c, which determines their ability to become electrically polarized (i.e., separate positive and negative electrical charges). The higher dielectric constant materials increase charge storage capacity, and the lower dielectric materials could be useful in electronic circuits. The complex dielectric constants measured for MAPbBr_3_ (0.6 mm) and MAPbI_3_ (0.8 mm) lie between 1.02–7.72 and 1.10–4.57, respectively, which are even higher than that of their inorganic counterpart CsPbBr_3_ [29] and conventional perovskite materials such as SrTiO_3_ and Cs_3_Bi_2_I_9_ [15,30]. This indicates that MAPbBr_3_ and MAPbI_3_ are promising materials for charge storage applications and can modulate the performance by simply varying the sample thickness. The absorption coefficient α (cm^−1^) was measured based on the extension coefficient; the obtained values and peak positions are shown in Figure 10b for MAPbBr_3_ and Figure 11b for MAPbI_3_. In the present work, it was achieved that reported PSCs almost possess similar absorption peak positions with slight variations. For this, we compared the obtained experimental absorption/conductivity peak positions with earlier reported works, which are summarized in Table 1.

In the above-mentioned table of earlier works, researchers demonstrated the THz vibrational modes for various thin films of MAPbBr_3_ and MAPbI_3_. From these measurements, the similarities found in MAPbBr_3_ andMAPbI_3_ are the following: (1) the SCs possess nearly similar frequencies due to chemical similarity of the compounds; (2) the higher thickness leads to a small blueshift in the absorption positions; and (3) in both PSCs, higher values of measured optical parameter properties were achieved for smaller thicknesses. On the other hand, these two crystals have some differences in their optical and morphological properties. MAPbBr_3_ possesses a cubic crystal structure, whereas MAPbI_3_ has a tetragonal crystal structure. In the Section 3.1. we provided the details of structural and optical properties in UV-Vis NIR domain. However, regarding the THz radiation, the vibrational frequencies of the two crystals majorly pointed to around 0.8, 1.4, and 2.0 THz for MAPbBr_3_ and 1.05 and 2.0 THz for MAPbI_3_ PSCs. For example, Maeng et al. reported the theoretical simulation of the THz absorption spectra of cubic MAPbBr_3_ (Figure 10b, bottom panel) [19], and the peak positions are well matched for 0.3, 0.6, and 0.8 mm thick crystals (shown in Figure 10b, upper panel). In addition, they proved that three phonon modes originate from the transverse vibration (0.8 THz), the longitudinal optical vibrations (1.4 THz) of the Pb–Br–Pb bonds, and the optical Br vibration (2.0 THz) [19]. However, an increase in crystal thickness leads to a smaller blueshift of THz frequencies.

Similarly, for MAPbI_3_ PSCs, the absorption spectra shown in Figure 11b (upper panel, 0.8 mm MAPbI_3_) were fitted with Lorentz peaks at 0.35, 0.95, and 2 THz. The same Lorentz fits were also suitable for the other thicknesses of crystals (data shown in Figure 11b bottom panel). In the case of MAPbI_3_, the absorption spectra having a slight difference in their values for MAPbBr_3_ is due to changes in the phonon vibrations between Pb-I and Pb-Br. Here, one the limitation of our study is that we could not calculate the bond angles and bond lengths of Pb-I-Pb or Pb-Br-Pb to reveal the exact vibrational frequencies and reasons causing their shift with respect to the thickness of the PSCs. However, most of the theoretical and experimental investigations revealed that the peak at 0.95 THz in MAPbI_3_ was mostly due to a Pb–I–Pb rocking vibration, and another peak at 1.89 THz corresponded to a Pb–I stretching vibration. In addition, in the MAPbI_3_ SC case, earlier researchers measured the conductivity in the terahertz domain, and subsequent comparisons are made below. As per Equation (9), the dark conductivity of the sample majorly depends on thickness, refractive index, and transmittance of the SC, whereas the absorption coefficient primarily depends on the imaginary refractive index. Thus, the peak positions for absorption spectra and dark conductivity in the case of both PSCs are almost identical curves, as shown in Figure 8b,c. The peak positions of THz frequencies are listed in the corresponding graphs. In the case of dark conductivity for MAPbI_3_ in the 0.5 to 2.0 THz range, the peak positions achieved at 1.05 THz and 1.95 THz are due to the fastening of the Pb–I–Pb angles and the Pb–I bond vibration, respectively, as per Ref [18,33].

**Figure 12:**
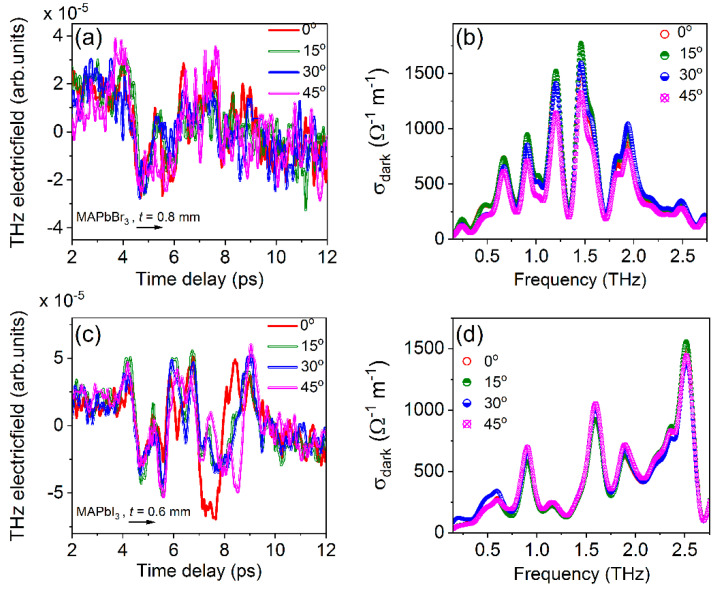
(**a**) THz electric field and (**b**) measured dark conductivity of 0.8 mm thick MAPbBr_3_; (**c**) THz electric field and (**d**) measured dark conductivity of 0.6 mm thick MAPbI_3_ for the incidence angle THz pulses between 0 and 45°.

Figure 12a,c show the transmitted THz electric field of MAPbBr_3_ (0.8 mm) and MAPbI_3_ (0.6 mm) for the incidence angles of THz pulses between 0 and 45^o^, respectively (the schematic for the rotation of these crystals shown Figure 2d). As we mentioned earlier for these samples, we used the reference pulse 2, shown in Figure 8a. The temporal profiles shown in Figure 12a,c seem to have similar tendencies with respect to different angles of incidence of THz pulses to the PSCs’ surfaces. We measured the dark conductivity of these PSCs, as shown in Figure 12b,d, for MAPbBr_3_ and MAPbI_3_, respectively. Interestingly, a small change in conductivity values was achieved without effecting their phonon vibrations for 0, 15, 30, and 45^o^ angles of incidence of THz pulses to the PSCs’ surfaces. As shown in Figure 12b,d, for MAPbBr_3_/MAPbI_3_ the peak positions are exactly the same with respect to different angles of incident and possess little variation in the peak values. This indicates that the phonon vibrational frequencies are independent of the incident angle of THz pulses to the crystals’ surfaces. All other different-thickness PSCs studied in this work also possess a similar tendency, i.e., the angle of incidence does not affect their phonon vibrations.

In the case of the MAPbBr_3_ polycrystalline thin film, Inhee Maeng et al. reported the THz wave absorption measurement and verified it with first-principles simulation (cubic MAPbBr_3_); three phonon modes originate from the transverse vibration (0.8 THz), the longitudinal optical vibrations (1.4 THz) of the Pb–Br–Pb bonds, and the optical Br vibration (2.0 THz) [19]. In the present case, the MAPbBr_3_ SC possess similar absorption peaks. In addition, the THz absorption spectra of MAPbBr_3_ SC are similar to the earlier theoretical simulation performed by Inhee Maeng et al. [19]. Similarly, for MAPbI_3_ PSCs, the absorption spectra having a slight difference in their values for MAPbBr_3_ is due to changes in the phonon vibrations between Pb and I compared to Pb and Br. However, for the MAPbI_3_ SC case, the earlier researchers measured the conductivity in the terahertz domain, and subsequent comparisons are made below. As per Equation (9), the dark conductivity of the sample majorly depends on thickness, refractive index, and transmittance of the SC, whereas the absorption coefficient primarily depends on the imaginary refractive index. Thus, the curves shown in Figure 8b,c are almost identical. The peak positions of THz frequencies are listed in the corresponding graphs. In the case of dark conductivity for MAPbI_3_ in the 0.5 to 2.0 THz range, the peak positions achieved at 1.05 THz and 1.95 THz are due to the fastening of the Pb–I–Pb angles and the Pb–I bond vibration, respectively [18,33].

Further, there is a scope to extend this work to find the conductivity and mobility of bulk PSCs. Additionally, we need optical pump excitation with delay dependence between THz probes. Currently, due to the limitation of the longer delay stage of the experiments, we could not perform the optical pump–terahertz probe experiments. In the future, we can exclusively elaborate THz optical properties of various-thickness PSCs using different optical pumps (800 nm and 400 nm wavelengths) using time-resolved THz-TDS in transmission and reflection geometries. As per our knowledge, the main limitation of this work is that we did not perform the THz-TDS in vacuum or nitrogen purging environments. Even though we generated THz pulses from air plasma, there is a scope of nitrogen purging/creation of a vacuum before PM1 (after air plasma) and after ZnTe (THz detection of crystals). However, primarily due to the lack of vacuum chambers, we performed our THz-TDS system in an ambient environment. The water content in the atmosphere may lead to the absorbing of the THz radiation, and as a result one can see the reference pulse amplitude (shown in Figure 8b) has some losses around 1–3 THz. However, we used the same reference pulse for all studied single crystals with varying thicknesses. Therefore, the obtained THz parameters are valid. In the future, to avoid the losses of amplitudes of THz pulses, we could initiate our experiments within a nitrogen purging environment.

## 4. Conclusions

We successfully synthesized different thicknesses of MAPbBr_3_ and MAPbI_3_ bulk PSCs using the ITC method. The measured steady-state absorption properties, photoluminescent emissions, and crystallinities in the case of 0.6 mm MAPbBr_3_ and 0.8 mm MAPbI_3_ are in good agreement with earlier reported works. In addition, we exposed the synthesized PSCs to THz-TDS and performed our experiments with two sets of the THz pulses generated from air plasma. In the first set, we chose the thickness of MAPbBr_3_ as 0.6 mm and MAPbI_3_ as 0.8 mm, and for the second set 0.3 and 0.8 mm for MAPbBr_3_ and 0.6, 0.9, 1.3, and 2.3 mm for MAPbI_3_. However, both reference pulses seem similar to each other in their temporal form. The measured transmittance based on the orientation of BBO (for generating THz pulses) implies that PSCs’ transmittance also follows the same tendency. In addition, PSCs’ azimuthal orientation and movement along the Z-path at optimized reference pulse provide that the transmittance (THz optical properties) is independent of these parameters. Further, we elucidated the response of THz optical parameters based on different thicknesses of PSCs and angles of incidence of the reference THz pulse to the crystal surface. These measurements show that the smaller thicknesses of PSCs possess higher values of the refractive index, dielectric constants, absorption coefficients, and dark conductivity, even though the PSCs transmit a higher amount of THz pulses. For example, the 0.3 mm MAPbBr_3_/0.6 mm MAPbI_3_ possess a real refractive index between 1.55–3.35/0.18–3.17 with average values of 2.09/2.25 in the 0.1 to 3.0 THz range. The increased values of the THz optical parameters in the case of the smaller thicknesses reveal the reliability of PSCs’ THz transmission. Meanwhile, the peak positions (THz frequencies) of absorption/dark conductivity show a slight blueshift with respect to the increase in the PSCs’ thicknesses, whereas the incident angles of THz pulses on the single crystal surface do not affect the phonon vibrations (even no small blueshift is observed). Overall, the present study provides insights on the possibility of a suitable thickness of MAPbBr_3_ and MAPbI_3_ PSCs for the maximum transmission of THz radiation and their potential applications in the THz domain.

## Figures and Tables

**Table 1:** Literature review for phonon vibrations for thin films and single crystals of MAPbBr_3_ and MAPbI_3_.

Materials/Methods	Regime (THz)	Each Row Represents Peak Positions of Phonon Vibrational Frequencies in THz								Ref.
MAPbBr_3_ thin films										
Theory	0.1-5.0	0.3	0.45	0.81	0.9	1.35	1.53	2.19	5.01	[31]
Theory/Experiment	0.25-3.0						1.5	2.0		[32]
Theory	0.25-2.75					1.3		2.25		[16]
Theory/Experiment	0.25-2.5			0.8		1.4		2.0		[19]
MAPbBr_3_ single crystals										
Theory/Experiment	0.25-2.5	0.2	0.59	0.71	0.93, 1.26	1.35	1.84	2.11, 2.29		[19]
Experiment	0.1-3.0	0.38	0.53		1.05, 1.21	1.58	1.88	2.11 2.34		This work
MAPbI_3_ thin films										
Theory/Exp	0.25-2.6				0.95	1.58	1.87			[20]
Experiment	0.3-1.0			0.7						[14]
Theory	0.25-3.0				1.0			2.0		[16]
Theory/Experiment	0.25-3.0				1.05			2.25		[33]
Theory/Experiment	0-2.25				1.0	1.6		2.0		[34]
Theory/Experiment	0.5-2.5				1.0			2.0		[35]
Theory/Experiment	0.25-3.0				0.95		1.92		2.7	[36]
Experiment	0-3.0				1.0			2.0		[37]
Experiment	0.1-3.0	0.37			1.05	1.28	1.95	2.11	2.23	Thiswork

## Data Availability

The data provided in the manuscript can be made available from the corresponding authors upon reasonable request.
